# Parallel molecular routes to cold adaptation in eight genera of New Zealand stick insects

**DOI:** 10.1038/srep13965

**Published:** 2015-09-10

**Authors:** Alice B. Dennis, Luke T. Dunning, Brent J. Sinclair, Thomas R. Buckley

**Affiliations:** 1 Landcare Research, Private Bag 92170, Auckland, New Zealand; 2 Allan Wilson Centre, Auckland, New Zealand; 3 School of Biological Sciences, University of Auckland, Auckland 1142, New Zealand; 4 Department of Biology, The University of Western Ontario, London, ON N6G 1L3, Canada

## Abstract

The acquisition of physiological strategies to tolerate novel thermal conditions allows organisms to exploit new environments. As a result, thermal tolerance is a key determinant of the global distribution of biodiversity, yet the constraints on its evolution are not well understood. Here we investigate parallel evolution of cold tolerance in New Zealand stick insects, an endemic radiation containing three montane-occurring species. Using a phylogeny constructed from 274 orthologous genes, we show that stick insects have independently colonized montane environments at least twice. We compare supercooling point and survival of internal ice formation among ten species from eight genera, and identify both freeze tolerance and freeze avoidance in separate montane lineages. Freeze tolerance is also verified in both lowland and montane populations of a single, geographically widespread, species. Transcriptome sequencing following cold shock identifies a set of structural cuticular genes that are both differentially regulated and under positive sequence selection in each species. However, while cuticular proteins in general are associated with cold shock across the phylogeny, the specific genes at play differ among species. Thus, while processes related to cuticular structure are consistently associated with adaptation for cold, this may not be the consequence of shared ancestral genetic constraints.

Variation in thermal tolerance drives global patterns of species richness, enables geographic range expansions, and contributes to species diversification^1^,^2^. At the molecular level, adaptive phenotypic changes, such as novel temperature tolerance, are achieved through both structural and regulatory modifications^3^,^4^, with the latter allowing for adaptive change in genes whose sequence is functionally constrained^3^. Among closely related species, repeated invasion of the same challenging environment suggests an underlying predisposition for adaptation, and study of such phenotypic parallelism provides an avenue to investigate both the genomic basis of adaptation and the constraints imposed by both the available genetic variation and underlying biochemistry^5^,^6^,^7^. Evidence from a number of systems^8^,^9^ suggests that such parallel phenotypic evolution is more likely to utilize the same genes and genomic regions, but much of this evidence comes from studies of closely related species and ecotypes in which diverging populations begin with the same standing genetic variation (e.g. repeated freshwater invasion in sticklebacks^10^ and pigment loss in cave fish^11^). Less is known about the role of genetic constraints in deeper evolutionary time or the evolution of conserved biological functions. Here we explore the physiological and genomic basis of cold adaptation in New Zealand (NZ) stick insects, a group that has been radiating for approximately 30 Myr.

The physical and biochemical impacts of low temperatures mean that cold is a significant determinant of insect performance and geographic distribution^12^. Below 0 °C, there is a risk of ice formation, and insects that survive below this temperature do so by either withstanding internal ice formation^13^ (freeze tolerance or partial freeze tolerance) or utilizing cryoprotectants to maintain the body fluids in a liquid state^12^ (freeze avoidance). Freeze tolerance and freeze avoidance are alternative strategies^14^ but share many biochemical mechanisms^15^. Freeze avoidance is likely the ancestral trait in terrestrial arthropods^16^,^17^, but freeze tolerance has arisen independently in many insect orders^17^, and its biochemical correlates (such as polyol cryoprotectants and antifreeze proteins) have received considerable attention^15^. While freeze tolerance is thought to be favoured in environments with prolonged periods of intense cold or variably cold conditions^17^, neither its mechanisms nor evolutionary origins are well understood. In particular, it is not known if the mechanisms of freeze tolerance are constrained within related species that have independently evolved the same strategy.

NZ stick insects are an endemic radiation of 23 described species derived from at least one ancestor that arrived from tropical New Caledonia (21°S) 26–30 Mya^18^ and are now distributed across the North and South Islands of NZ (34°S–47°S), where they feed on a range of native and introduced plants^18^. The ten NZ genera include both geographically restricted and broadly distributed lowland species (Fig. 1 and Supplementary Table 1), which are inferred to have contracted to warmer more northern habitats during the last glacial maximum^19^,^20^. Among these species, multiple comparisons of temperature tolerance and gene expression can be made. Although phasmids are usually warm-climate specialists, reaching peak diversity in lowland tropical forests^21^, members of three NZ genera have invaded montane habitats (at or above the tree line). *Niveaphasma annulata* and *Tectarchus salebrosus* both occur from sea level to approximately 1000 m a.s.l.^22^,^23^, while the genus *Micrarchus* contains both the lowland *M. hystriculeus* and the montane specialist *M*. nov. sp. 2 (found only between 650–1400 m a.s.l.^23^,^24^). All life stages of these montane-occurring genera, including both high- and low-land populations, overwinter in microhabitats where they experience freezing temperatures^25^. The breadth of environments occupied by these species, including multiple species occurring in cold southerly and montane habitats, make NZ stick insects an ideal group within which to study the basis of adaptation to low temperature and its variation.

Here we use wild populations to examine physiological and transcriptomic variation in cold tolerance among ten species belonging to eight genera of NZ stick insects, including six separate populations of a single, geographically widespread species. We measure physiological response to the cold and gene expression following cold shock, and map these responses onto a robust phylogeny. Our results demonstrate that at least two lineages have independently colonized montane and cold southern (high latitude) environments, and that these genera possess greater abilities to survive freezing events than lowland-restricted species. Transcriptome profiling of 12 populations from eight genera and ten species identifies both regulatory and structural changes in genes encoding proteins putatively associated with cuticle structure. This is the only functional gene category that is consistently associated with cold exposure in all taxa, and suggests that cold tolerance in NZ stick insects may, at least partially, require modifications in the protein sequence and expression of these structural cuticular genes. Interestingly, we find that different individual cuticular genes are implicated in each lineage (via either differentially expressed or evolving under positive selection). This suggests that, despite their shared genetic history, the repeated utilisation of these genes may be convergent on a common gene function, rather than on reusing the same genes due to ancestral constraints.

## Results and Discussion

### Phylogenetically independent montane colonisations in NZ stick insects

**Figure 1:**
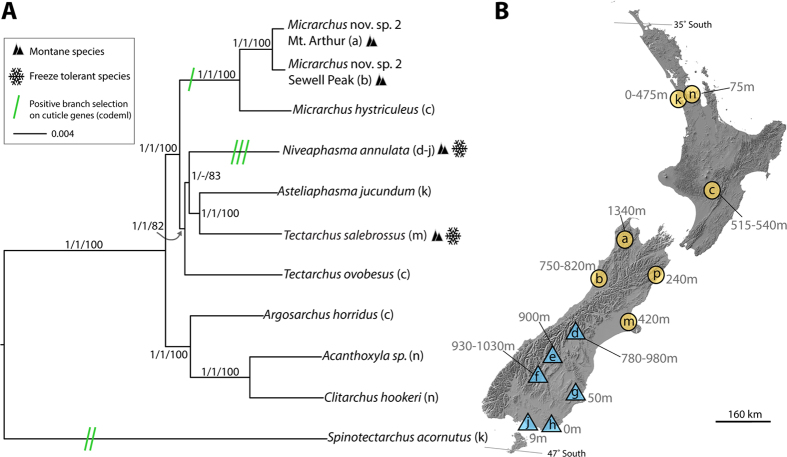
(**A**) Consensus phylogeny constructed using 274 conserved orthologs obtained from transcriptome sequences (200,279 bp). Support values are: nucleotide Bayesian posterior probabilities/amino acid Bayesian posterior probabilities/nucleotide maximum likelihood bootstrap values. The single node not reconstructed in the Bayesian amino acid analysis is indicated by “−”. Snowflakes designate species that demonstrated freeze tolerance and black triangles indicate species whose range includes montane habitat. Green bars indicate instances of cuticular genes in which positive selection was detected by branch-site models (see also Fig. 3). Parenthetical letters indicate collection locales marked on the New Zealand map (**B**) and in Supplementary Table 2. (**B**) NZ map showing collection locales (Supplementary Table 2) marked by blue triangles (*N. annulata*) and orange circles (all other species): (**a**) Mt. Arthur, (**b**) Sewell Peak, (**c**) Paengaroa, (**d**) Ohau, (**e**) Nevis, (**f**) Remarkables, (**g**) Dunedin, (**h**) Papatowai, (**j**) Seaward Moss, (**k**) Waitakere Ranges, (**m**) Port Hills, (**n**) Auckland, and (**p**) Puhipuhi.

To reconstruct the phylogenetic relationships among our study species, we used a pre-defined gene set to extracted >2,000 conserved, orthologous genes from the transcriptomic data used to compare expression following cold shock (Fig. 1, 11 datasets representing 10 species, see below). After filtering the results to remove sequences with missing data, we retained 274 genes that were at least 98% complete across all taxa. These totalled 200,379 bp and contained 20,936 variable sites (3,648 non-synonymous and 17,288 synonymous changes), which were concatenated for three separate phylogenetic analyses. All but one node were identically reconstructed across analyses (Fig. 1). Nodal posterior probabilities of 1.0 (Bayesian analyses of nucleotide and amino acid sequence) or likelihood bootstrap values of 100% were estimated in most cases. The identical Bayesian nucleotide and likelihood trees were used in subsequent analyses.

In the resulting phylogeny (Fig. 1), montane lineages were not monophyletic with respect to lowland lineages. This suggests that speciation into the montane environment has occurred two or three times in NZ stick insects, with *Micrarchus* as an independent lineage containing the montane specialist *M.* nov. sp. 2, and *N. annulata* and *T. salebrosus* representing another one or two colonisation events. *Spinotectarchus acornutus* is also deeply divided from all other species, suggesting that it either arrived in NZ from New Caledonia in a separate colonisation event or is the result of a very early division from the ancestral species that founded all other NZ species^18^,^26^.

### Independent montane lineages employ different strategies to survive freezing

**Figure 2:**
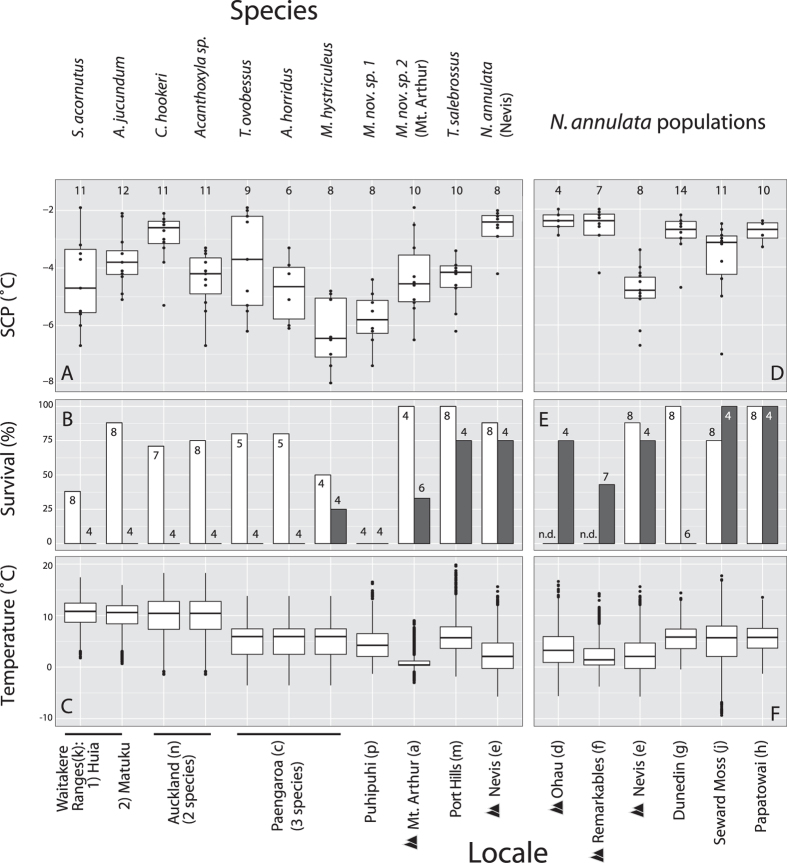
Cold tolerance and microclimate temperatures for 11 species of New Zealand stick insects (A–C) and six populations of *N. annulata* (D–F). Letters next to locale labels on lower x-axis correspond to map locations in Fig. 1B. (**A**,**D**) Supercooling point (SCP); solid middle line of the box plots indicates the median and the box the interquartile range of measurements. Whiskers extend to extreme data points within 1.5 of the interquartile range, and all data points are plotted. Sample sizes are printed above boxplots (**B**,**E**) Survival following a small amount of internal ice formation at the SCP (white bars) and 6 h frozen (grey bars). Sample sizes listed above/within bars, and (**C**,**F**) Microhabitat temperature of three coldest months of the year (June-August) for each collection site. Box plots are as in (**A**,**C**); only extreme data points have been plotted. Black triangles indicate montane collected populations and “n.d.” indicates populations for which SCP survival was not measured (in these cases SCP measures come only from the 6 h frozen treatment).

To compare cold physiology among 11 species, we examined cold tolerance by measuring supercooling point (the temperature at which ice spontaneously forms in the body, SCP), survival after cooling to this SCP, and survival of internal ice for six hours (6 h) (Fig. 2, Locales and collections in Fig. 1 and Supplementary Table 2). Together, these measures allow us to identify whether a species is killed at or before the SCP (chill susceptible or freeze avoidant^27^), is freeze tolerant (survives significant conversion of body water into ice^12^), or is only partially freeze tolerant (survival at the SCP; tolerates a small amount of internal ice^13^). All animals were collected in the field near the end of the austral summer and tested after a short acclimation period in the lab. Since most species cannot be successfully reared in controlled environments, this was the only way to collect sufficient individuals for our tests. Although the SCP is phenotypically plastic in many species^28^, the relatively warm SCPs we observe are indicative of the presence of ice nucleating agents in the hemolymph or gut, as is typical of feeding insects^29^. Diet can be a significant determinant of SCP in arthropods^30^, so it is possible that any similarity of SCPs is due to a common diet, but simply modifying the SCP with exogenous nucleators is insufficient to modify the cold tolerance strategy^16^. Furthermore, many of our sites experienced burst of cold weather, including freezing events, in the weeks immediately following our collections^25^, leading us to expect that insects from these locales would have been physiologically prepared for cold exposure at the time of collection.

Across all species, SCP ranged between −1.7 °C and −8.0 °C (Fig. 2, values reported in Supplementary Table 3). SCP was lowest (i.e. coldest) in two of the three *Micrarchus* lineages tested (the lowland *M. hystriculeus* and *M.* nov. sp. 1), followed by the remaining *Micrarchus* (the montane *M.* no. sp. 2), *S. acornutus* and *Acanthoxyla*. Among all species tested, only the lowland *Micrarchus* and northerly distributed *S. acornutus* had low (<50%) survival (measured for 1 week) following the small amount of internal ice formation at the SCP (Fig. 2), suggesting that they are either chill susceptible or freeze avoidant. In contrast, all other species tested had >70% survival after reaching their SCP. This demonstrates an underlying ability to survive some internal ice formation that has likely facilitated the diversification of stick insects, apart from the *S. acornutus* lineage, in temperate NZ in spite of their tropical ancestry^18^, and suggests that this ability may be lost in some of the *Micrarchus* lineage.

Surviving the partial formation of internal ice is likely insufficient to successfully overwinter in the cold lowland and montane environments occupied by *N. annulata*, *T. salebrosus* and *M.* nov. sp. 2, where our data loggers regularly measured freezing across several the winter months^25^ (Fig. 2 and Supplementary Table 4). In our tests of survival following 6 h of internal ice formation, high proportions (>50%, Fig. 2 and Supplementary Table 3) of both *N. annulata* and *T. salebrosus* survived. *Niveaphasma annulata* generally froze at higher sub-zero temperatures (−1.7 °C to −7 °C, Fig. 2) than the winter lows recorded at our collection sites (1 °C to −11.6 °C, Fig. 2), strongly supporting freeze tolerance as the overwintering strategy for this species. The SCP of *T. salebrosus* (−4.5 °C) was lower than the minimum microhabitat temperature at its collection site (−2.5 °C), suggesting it does not freeze at this locale; however the 420 m a.s.l. collection locale (Port Hills) likely does not represent the coldest habitats occupied by *T. salebrosus*, which has been collected above the tree line elsewhere (Records from New Zealand Arthropod Collection, Landcare Research, Auckland, New Zealand). Thus, both geographic distribution and survival following 6 h frozen suggest that *T. salebrosus* may also survive sub-zero conditions via freeze tolerance.

By contrast, *Micrarchus* species do not appear to be freeze tolerant. *Micrarchus* species had the lowest SCP of all the species examined, relatively high mortality after brief ice formation at the SCP, and low survival following 6 h of internal ice formation (<35% in *M. hystriculeus* and *M.* nov. sp. 2, 0% in *M.* nov. sp. 1). The SCPs of *M*. nov. sp. 1 (−6.6 °C) and *M. hystriculeus* (−6.3 °C) are also lower than the minimum temperatures recorded at their collection sites (−2.5 °C and −5.5 °C respectively, Fig. 2) suggesting that they do not normally experience ice formation in the field. Additionally, *Micrarchus* individuals all survived the −5 °C cold shock prior to RNA-seq (below), which suggests they are not killed by cold exposure in the absence of ice formation. Thus, we propose that *Micrarchus* species survive low temperatures in their habitat by supercooling and avoiding internal ice formation. Only the montane *M.* nov. sp. 2, which we sampled from Mt. Arthur, had a SCP (−3.2 °C) above the minimum habitat temperature (−5.5 °C), but its relatively low survival of 6 h ice formation makes it difficult to conclude that it is freeze tolerant in the field. Mt. Arthur has consistent and deep snow cover, likely buffering temperatures such that *M*. nov. sp. 2 remains unfrozen through most of the winter (Fig. 2,^25^,^31^). Thus, despite the apparent freeze avoidance in *Micrarchus*, the varied responses across these species may also reflect the high levels of genetic subdivision and greater habitat specialty found in *Micrarchus*^23^,^24^, warranting further investigation.

In contrast to most other NZ stick insects, *S. acornutus* had a mean SCP (−4.4 °C, Fig. 2 and Supplementary Table 3) lower than its minimum habitat temperature, and very limited survival of internal ice formation; this is the only other species that did not show relatively high survival following SCP measures. This inability to deal with sub-zero temperatures is likely associated with its restriction to the forests of subtropical northern NZ, where it experiences very few freezing events, and those that occur may be readily buffered by microhabitat selection^25^. This lack of cold tolerance may explain why *S. acornutus* has not diversified into cooler, more southerly habitats.

Thus, at least two lineages of NZ stick insects have diversified into freezing habitats during the course of their radiation, probably utilizing two different cold tolerance strategies: freeze tolerance (*N. annulata* and *T. salebrosus*) and freeze avoidance (*Micrarchus*). Our observations are consistent with tropical niche conservatism^32^, whereby global biodiversity patterns are driven by the acquisition of traits required for non-tropical environments. This hypothesis predicts that acquisition of key traits (such as freeze tolerance) must accompany range expansion into cooler habitats, and that the rarity of such trait evolution limits the number of species dispersing poleward, and leads to decreasing species diversity with increasing latitude^32^. We observe several aspects of this process here: (1) Partial freeze tolerance associated with SCP measures shows that NZ stick insects can presumably tolerate cooler temperatures than their tropical New Caledonian ancestors, (2) Relative to the more distantly related *S. acornutus*, all other sampled NZ lineages have a greater ability to tolerate rare freezing events (via freeze tolerance or supercooling) and most are distributed farther south, and (3) just three lineages have evolved the greatest tolerance of sub-zero temperatures: via supercooling in *Micrarchus* and freeze tolerance in *N. annulata* and *T. salebrosus*), and these lineages occur in the coldest southern and montane habitats.

### Freeze tolerance in six populations of *Niveaphasma*

Despite its potential contribution to species diversification and the evolution of novel traits, among-population variation in thermal tolerance has been poorly explored in insects outside of *Drosophila*^33^. We measured SCP and survival of 6 h ice formation in three lowland and three montane populations of the freeze tolerant *N. annulata*, including two parthenogenetic montane populations (Remarkables and Nevis; Fig. 2 and Supplementary Table 3). SCP was more consistent and warmer in the coldest locales including high altitude populations (Ohau, Nevis, and Remarkables, Figs 1 and 2) and the most southward exposed lowland site (Seward Moss). Both montane and lowland populations had relatively warm supercooling points and high survival following 6 h frozen (Fig. 2 and Supplementary Table 3). Although *N. annulata* does not show strong geographical population structure, this observed freeze tolerance spans the three known mitochondrial DNA clades^22^, suggesting that phylogeographic structure (ancestral genetic constraint) does not explain the variation in freeze tolerance we observed within this species. The only exception to this was the lowland Dunedin population, which did not survive 6 h ice formation (Fig. 2) and had a slightly depressed supercooling point. This was the warmest of the *Niveaphasma* sites (Fig. 2), hinting that there may be variation among locales in cold response. Alternatively, individuals from Dunedin were smaller than at the other sites, suggesting that they may not have been as mature as individuals from the other populations. It is possible that adults of this species are more cold-tolerant than juveniles, and we therefore failed to capture this developmental plasticity in our study.

### Differential gene expression in ten species

To investigate the transcriptomic basis of cold tolerance in NZ stick insects, we compared gene expression among 12 populations representing ten species (Table 1). This included the ten species comprising the tree in Fig. 1, two populations of *N. annulata* (lowland Dunedin and montane Remarkables), and two populations of *M.* nov. sp. 2 (which only occurs in montane locations: Mt. Arthur and Sewell Peak). Only *M*. nov. sp. 1 was included in physiology experiments above and not transcriptome comparisons. As with the phenotypic comparisons, we used wild caught individuals collected in late summer. Within each species or population, we compared expression between three cold shocked (1 h at −5 °C, followed by 1 h recovery) and three control individual stick insects. This same experimental design was used for each of the species tested, except for *M.* nov. sp. 2 from Mt. Arthur, where we increased sample sizes to six cold shocked and six control individuals. This treatment was chosen because of its potential to induce rapid expression of transcripts associated with brief, non-lethal cold shocks that may be associated with both recovery and enhanced cold tolerance in subsequent exposures^34^,^35^. By including a full hour of recovery, we could ensure that all species were moving in a coordinated fashion and had survived the treatment, and the results could also be better compared to previous studies^23^,^24^,^36^,^37^,^38^,^39^.

Illumina sequencing produced 39.4 Gb, or 3–5 Gb of data *per species* (n = 6–12, Data and assemblies summarized in Supplementary Table 5, Raw reads deposited in SRA, BioProject: PRJNA231979). Each species was assembled and analysed separately, as there is not a suitable reference genome available. The two populations of *M.* nov sp. 2 were analysed separately throughout because they possess different mitochondrial DNA backgrounds^23^. The two populations of *N. annulata* were combined into a single *de novo* assembly and then analysed separately. In total, there were 11 *de novo* assemblies representing datasets from 10 species and 12 separate locales (Table 1, Full collection details in Supplementary Table 5). The number of reads uniquely mapping back to each transcriptome was generally high (63.3–96.4%, mean of 87.6%). Between 269 and 9348 transcripts were significantly down-regulated and 315–8143 transcripts were significantly up-regulated after cold shock in each species; variation in both mapping efficiency appears to be due to higher fragmentation in some *de novo* assemblies, rather than a biological differences between samples. Significance was determined by combining the results of three statistical packages (edgeR^40^ p < 0.05, DESeq^41^ p < 0.10 and baySeq^42^ Li > 0.70, Table 1, Results from each package is given in Supplementary Data 1).

We examined each dataset for a set of *a priori* genes associated with the cold in other insects. Based on the best blast hits of our transcripts, we examined expression in genes associated with carbohydrate metabolism^36^, heat shock proteins^37^, homologs of smp-30^38^, 14 different genes associate with cold hardening, cold shock and cold acclimatization in *Drosophila*^39^, and matches to “cold” and “freezing”. In all, we examined expression in 37 genes, not including heat shock proteins. Of these, 24 were present in our transcriptiome data, and up to 82 transcripts matched heat shock proteins (and their associated chaperones, full results in Supplemental Data 2). While there were no overall patterns of gene expression strongly associate with phylogeny or physiology, we can make several interesting observations. Genes blasting to *smp-30* homologs were expressed in all datasets, and significantly differentially expressed in three (up-regulated in *C. hookeri* and *M.* nov. sp. 2, down-regulated in *N. annulata* from Dunedin). Homologs to this gene are expressed in cold acclimated *Drosophila* (15 °C), are proposed to function to maintain cytosolic Ca^2+^ levels at low temperature, and may be associated with stress response elements in yeast and plants^38^. Thus, this suggests some common response to the cold between stick insects and the better studied, but largely chill susceptible, *Drosophila*.

In addition to five candidate carbohydrate metabolism genes identified by Waagner *et al.*^36^, we also searched for “carbohydrate” in our transcripts. This yielded carbohydrate sulfotransferase, a widely functioning molecule, some forms of which may have metabolic function^43^. Including this, we find three different transcripts that are significantly up-regulated in *M.* nov. sp. 2 from Sewell peak, as well as five in the *S. acornutus*. This shows parallels between gene expression in these taxa (which we have identified as freeze avoidant and freeze intolerant, respectively), and the source of these genes: the freeze intolerant springtail *Folsomia candida*, which up-regulated these genes following rapid cold hardening (1 h at 0 °C). These transcripts were not up-regulated in any other taxa, but were down-regulated in the lowland *M. hystriculeus* (1 transcripts), *A. jucundum* (1 transcripts), and *N. annulata* Dunedin (the lowland population, 2 transcripts), the latter two of which have shown higher tolerance of ice formation at the SCP (Fig. 2). These proteins are thought to aid in protein stabilization and increase survival in the cold, even in the absence of ice formation^36^, This suggests they may play a similar role here, primarily in non- freeze tolerant stick insects.

Among the suite of cold-associated genes identified in *Drosophila*^39^, we identified only two with significant differential expression. The first of these, *Pyrroline 5- carboxylate reductase* (one copy down-regulated in *C. hookeri* and two copies down-regulated in *N. annulata* from Dunedin) is important in proline synthesis and points to this pathway as important in stick insect cold tolerance. The second, *Cytochrome b5-related* (one copy up-regulated in *S. acornutus*, one in *M.* nov. sp. 2 from Sewell) is part of lipid metabolism and was down-regulated both here and in *Drosophila*. In both cases, the direction of regulation is the same as that found in the relatively cold intolerant *Drosophila*, and further supports that these species may not tolerate freezing. Our searches for “cold” and “freezing” among the expressed genes yielded (respectively) a putative cold-shock domain and antifreeze protein that have been primarily characterized in vertebrates; the only differentially expressed genes among these was a cold-shock domain that was up-regulated in *A. horridus* (Supplemental Dataset 2).

Perhaps unsurprisingly, several different heat shock proteins were expressed in all datasets (except *M.* nov sp 2 Mt Arthur). This included transcripts matching *hsp70*, which were differentially expression in seven datasets (*C. annulata*, *A. horridus*, *T. ovobessus*, *A. jucundum*, *N. annulata* Dunedin*, M. hystriculeus* and *M.* nov sp 2 Sewell). Notably, no transcripts matching to *hsp70* were differentially expressed two of the three montane collections (*N. annulata* Remarkables and *M.* nov. sp. 2 Mt. Arthur). Expression of *hsp70* is associated with the onset of hot or cold shock in *Drosophila*^37^, and this suggests that these two populations were less strongly affected by our cold shock treatment. Alternatively, missing transcripts in any of these datasets could simply be due to the stochasticity in this data, particularly since our analysis lacks a reference genome, so low coverage genes might not be included after removal of transcripts with poor coverage. As more genomic data from these taxa become available, further analysis of these genes in light of their orthology, particularly heat shock proteins, certainly warrants further investigation. These results also highlight that, although we sought to standardize treatments, there is likely variability among datasets. To minimise the effect of environmental variation prior to insect collection, we used common rearing conditions in the lab prior to treatment, and we collected as many species as possible from the same locale (Fig. 2 and Supplementary Table 2, Waitakere Ranges: 2 species, Paengaroa: 3 species, Auckland: 2 species). Furthermore, our previous work has also shown that these methods are able to reveal fixed differences among locales, even within species. For example, Dunning *et al.*^24^ used qPCR validation in *Micrarchus* to detect the same expression patterns between years.

**Figure 3:**
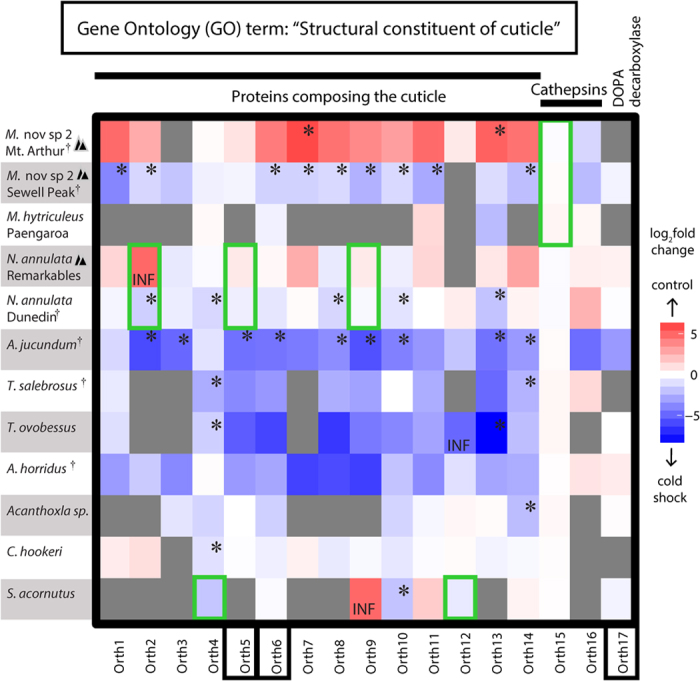
Heat map of log_2_ fold change (calculated in DESeq) of orthologous genes belonging to the *structural constituent of cuticle* GO category. Grey boxes indicate absent or low copy number genes. Asterisks (*) denote significantly differentially expressed genes. INF marks genes that were only expressed in that treatment; for these, log_2_fold change has been arbitrarily set at +5/−5 for colouring. Black boxes denote genes with amino acid sites under selection across the entire phylogeny (site models). Green boxes denote genes with amino acid sites under lineage specific selection (branch-site models). ^§^indicates species with significant differential expression of the GO category “structural constituent of cuticle” (Fisher’s exact test) and black triangles mark montane collected populations. Gene categories listed above the graph are based on best hit in the NCBI database using Blastx (Supplementary Data 2).

To compare enrichment of molecular functions, rather than individual differentially expressed genes, we performed a Fisher’s exact test using Gene Ontology (GO) categories^44^ separately assigned to each species based on their blast results in Blast2GO^45^. Within each species, this analysis returned between 0 and 624 GO terms significantly associated with cold shock (FDR < 5%, full results for each species in Supplementary Data 3). Only one GO category was repeatedly enriched across datasets: “Structural constituent of cuticle” (GO:0042302, ontology: Molecular Function). Genes from this ontological set were up-regulated in response to cold-shock in five species: *M.* nov. sp. 2 from Sewell Peak, *A. jucundum*, *N. annulata* from Dunedin, *T. salebrosus*, and *A. horridus*. In the latter three this was the only category that was significantly up-regulated. This GO term was also down-regulated in one dataset: *M*. nov. sp. 2 from Mt. Arthur, for which it was the only significant GO term, and it showed non-significant down-regulation in *N. annulata* from the Remarkables (Fig. 3). In *M*. nov. sp. 2 from Mt. Arthur (and to a lesser extent *N. annulata* from Remarkables), we postulate that these genes could perform the same function, but that their rate of expression (perhaps due to the evolution of regulatory factors) means they were no longer up-regulated when we sampled RNA one hour after cold shock^23^,^46^. Whether it is a difference in the speed of response, or in the overall difference in the expressed genes in these species, these results hint at potential plasticity in the cold shock responses among populations within species. It is important to note that only the annotated portion of our dataset was compared by this functional gene enrichment (~25% of transcripts >200 bp); as additional annotations become available, they will only build on this story^47^,^48^. Nonetheless, these results point strongly to a role of genes encoding the structure of the cuticle in the cold response of NZ stick insects.

### Positive selection in cuticle-associated genes

Based on the repeated observation of differential expression in structural cuticular genes, we hypothesized that selection may be acting on the protein coding sequence of these genes to confer adaptation to the cold. To investigate this we tested for selection on a set of orthologous genes belonging to this GO category using the codeml package in PAML^49^. We identified and aligned all orthologous structural cuticular genes (or partial genes) that were present in at least five datasets. Not all genes were significantly differentially expressed in our data (Fig. 3); we have labelled them Orth 1–17 (Fig. 3, calculated values in Supplementary Table 6). We tested for both amino acid sites under selection across the entire phylogeny (site models) and for branch-specific evidence of selection in just four branches (branch-site models): the branches leading to *N. annulata*, *T. salebrosus* and *Micrarchus* were tested for evidence of specific evolution associated with their montane adaptations, and the branch separating *S. acornutus* from all other species was used to test for evolution associated with greater cold tolerance in all other species relative to *S. acornutus.* Only branch-site tests with *T. salebrosus* as the foreground lineage did not detect branch specific positive selection (Green boxes, Fig. 3). This species has only recently been documented at altitude, thus further investigation of its range and the freeze tolerance observed here is warranted.

These “structural constituent of cuticle” genes can be grouped into three broad categories based on their best blast hits (Blast results are detailed in Supplementary Data 4). Most (Orth1–14, Fig. 3) are likely proteins that form part of the cuticle structure (“Proteins composing the cuticle”, Fig. 3). All 14 of these are significantly differentially expressed in at least one dataset, and two (Orth 5 and 6) are under positive selection across the entire phylogeny (Fig. 3). Of the six genes with significant branch-site results, five were structural cuticular genes: positive selection was detected in three genes (Orth 2, 5 and 9) on the branch leading to *N. annulata* and in two different genes (Orth 4 and 12) on the branch separating *S. acornutus* from all other NZ species (Figs 1 and 3). For these two lineages, these were the only cuticle related genes with significant results from the branch-site tests, suggesting that modification of genes associated directly with cuticle structure may be a prerequisite for both the freeze tolerance observed in *N. annulata* as well as the greater cold tolerance observed in all species relative to *S. acornutus*. Thus, it is possible that the evolution of diverse cold tolerance abilities in NZ stick insects is at least partially enabled by the evolution of cuticle structure.

A further two genes within this GO term are identified as Cathepsins (Fig. 3). Among these, Orth 15 shows evidence of positive selection on the *Micrarchus* branch and Orth 16 across the entire phylogeny (Fig. 3). Orth 16 was the only gene with a significant branch-site model result in the *Micrarchus* lineage, suggesting that these genes are associated with the putative freeze avoidance of this group (which contains the only alpine specialist, *M*. nov. sp 2). Cathepsins are cysteine proteases and have been implicated in both insect metamorphosis and moulting^50^ (although we have sampled only adult females). Thus the utilisation of these genes is consistent with the structural cuticular genes described above and suggests repair, remodelling or reinforcement of the cuticle in cold exposed stick insects. Such cuticle-related genes have not previously been explicitly identified as candidates for cold tolerance in other species, but revisiting the dataset of Zhang *et al.*^51^, we find that *Cathepsin 1* is slightly differentially regulated in response to cold exposure in *D. melanogaster*, suggesting this could be an overlooked result in other systems.

Lastly, among significantly differentially expressed genes, Orth 17 was identified as a *dopa decarboxylase* and was significantly differentially expressed in only *A. jucundum*. This gene can be associated with wound repair and immunity, as well as cuticle structure, and may hint at cuticular damage from cold exposure^52^. *Asteliaphasma jucundum* is restricted to lowland and warmer northern forests, suggesting that expression of this gene may be in response to damage caused by cold exposure not seen in more cold-hardy species; *dopa decarboxylase* is also slightly down-regulated after cold exposure in *D. melanogaster*^51^.

Jointly considering both sets of tests for selection, we find evidence that cuticle genes may be evolving under both positive selection on amino acid sequence and selection for varied levels of gene expression. Together, these results suggest that structural cuticular genes were associated with cold exposure, and that several disparate genes are under selection across the NZ stick insect phylogeny. We can compare the results of selection tests back to the expression levels of these genes: all of the “Proteins composing the cuticle” genes (Fig. 3) were significantly differentially expressed in at least one species, while the three genes in the remaining two categories were not differentially expressed. This suggests that it is expression of structural genes that might be a direct response to cold shock. Modification of the cuticle makes intuitive sense when dealing with sub-zero temperatures: innoculative freezing (internal ice formation that is catalyzed from outside the body^53^) is a real risk for exotherms. Thus, altering cuticle thickness, texture, or structure may make a difference for surviving freezing snaps, particularly in freeze avoiding species. It is also possible that selection on these genes reflects both the external cuticle and the cuticular genes that line the gut, which is susceptible to cold damage in many insects^52^. Additionally, the cuticle is deposited in a circadian rhythm, but we do not believe that our observations can be explained by circadian rhythms because (1) we time-matched our sampling as far as possible, and (2) we see both up- and down-regulation of these genes across taxa, including those that were simultaneously treated and thus should have been in identical portions of their circadian rhythm (Supplementary Data 2). Another alternative hypothesis, which we cannot presently discount, is that cuticle genes have been variously co-opted into other, cold-stress related function, as is common in stress related biochemistry^e.g^ 54. In summary, we propose that NZ stick insects undertake cuticular remodelling in response to cold shock, but the nature and consequences of this remodelling will require more in-depth investigation.

## Conclusions

We have revealed that New Zealand stick insects likely employ two different strategies to cope with sub-zero temperatures. *Micrarchus* nov. sp. 2 had low survival following prolonged periods of freezing, supercooled to lower temperatures, and its geographical range only includes high altitude habitat that is often covered with snow for prolonged periods. Thus, for this species the more common arthropod strategy of freeze avoidance has been employed. In contrast, *N. annulata* and *T. salebrosus* occur in both lowland and high altitude habitats and experience more frequent temperature swings and appear to be freeze tolerant; this is the first observation of freeze tolerance in Phasmatodea. For these species, adopting freeze tolerance may be a more suitable strategy to cope with, for example, the dehydrating effects of multiple freeze-thaw cycles. In both cases, winter lows are not as extreme as those studied in some other freeze-surviving arthropods, and the idea that such subtle habitat differences can lead to different strategies warrants further investigation.

Along with these varied physiological strategies, our comparisons of gene expression suggest that cold exposure elicits varying responses across the species we tested. This includes several transcripts that are expressed in patterns similar to several other, primarily freeze intolerant, species including heat shock proteins and genes underlying carbohydrate metabolism^36^,^37^,^38^. Physiological variation among species may also involve selective pressure on both the expression and amino acid sequence of cuticular genes. This adaptive evolution could be associated with the range expansion of NZ stick insects from a more tropically distributed ancestor. The evidence we find for bursts of positive selection in different cuticular genes suggests that cuticular evolution in NZ stick insects is driven by different genes across different species. This multi-gene result is in contrast with other systems in which parallel adaptive evolution has repeatedly employed changes in the same genes^8^, whether sifting through ancestral polymorphisms (e.g. freshwater invasion in sticklebacks^7^,^55^) or via independent mutations (e.g. flowering time in *Arabidopsis*^56^ and gene expression of bioluminescence in squid^57^). Indeed, parallel speciation in *Timema* (sister group to all other stick insects) is accompanied by both divergent gene regions that are unique to each species and regions that repeatedly diverge in parallel^58^. These results suggest that even with a shared history and the potential for repeated evolutionary scenarios, identical genetic changes may not be the basis of parallel phenotypic evolution, and “idiosyncratic outcomes^58^” may dominate the genomic course of adaptation.

## Methods

### Collections and field measures

Insects were collected at sites throughout NZ in 2011 and 2012 (Fig. 1 and Supplementary Table 1) and all tests used only adult females because some NZ species are parthenogenetic. Data loggers were placed at each site to record environmental temperature every 1.5 hr for at least one winter season (Supplementary Table 3 and Fig. 2). Where possible, loggers were placed both in the leaf litter at the base of the plant and 0.5–1.0 m above include potentially buffered temperatures in the litter^25^. However, these differences were not as large as temperature differences between sites, so these two sets of measurements have been averaged to give a general temperature at the locale. More details of temperature data collected at many of these sites are in Dennis, *et al.*^25^.

### Cold tolerance measurements: SCP and freeze tolerance

We examined cold tolerance by measuring supercooling point (SCP), survival at the SCP and survival of internal ice for six hours in 11 species (Fig. 2 and Supplementary Table 2). Prior to treatment, all insects were maintained in controlled conditions (18 °C under 13:11 h light:dark cycle) for a minimum of seven days and fed *Rubus sp*. *ad libitum*. SCP and survival of internal ice formation were measured by placing insects, in contact with a thermocouple, in glass tubes suspended in a chilled methanol-water mix cooled at 0.25 °C/min. The SCP was taken as the lowest temperature prior to the onset of the freezing exotherm (a measurable temperature rise upon ice crystal formation). Survival of equilibrium ice formation was assessed by cooling insects to the SCP and holding them at this temperature for 6 h before warming at 0.25 °C/min and recovering at room temperature; this duration was chosen because it can take several hours for ice formation to reach equilibrium in insects^59^. Survival was assessed daily for one week.

### Cold treatments and RNA sequencing

Prior to Illumina sequencing, all individuals were maintained in the same, controlled conditions for a minimum of two weeks prior to treatment. Cold treatments were conducted in a Sanyo MIR-154 incubator. Insects were cooled to −5 °C, held for 1 h, warmed to room temperature for a 1 h recovery and snap frozen at −80 °C. To avoid potential contamination from gut microbiota, RNA was extracted from each stick insect using only tissue from the head, prothorax, and antennae. Extractions were performed using Trizol reagent, and then further spin column purified using the RNeasy mini kit. Total RNA quality was verified on an Agilent 2100 Bioanalyzer, and quantified with a Nanodrop spectrophotometer prior to cDNA library preparation using the Illumina TruSeq RNA kit (v1 for *Micrarchus*^23^, v2 for all other species). Single-end 50 bp cDNA libraries were sequenced by Illumina HiSeq2000 at the High Throughput Genomics Core Facility at the Huntsman Cancer Institute, University of Utah, with 8–18 individuals per lane.

### Phylogeny construction

Raw Illumina data was processed to trim adaptors and low quality sequences (phred score <30)^60^, homopolymer stretches^61^, all reads containing N’s^62^, and all reads <25 bp; quality was assessed using FastQC^63^. Individual transcriptomes were *de novo* assembled separately for each species using Trinity r2012.10.05^64^; redundancy among the resulting transcripts was reduced using CD-HIT-EST^65^ and ribosomal sequences were removed using riboPicker^66^ followed by additional manual removal. Final assemblies were annotated using Blastx^67^ against the NCBI *nr* database (March 2013, 1e^−10^ cut-off). Each transcript was analysed separately from this point on (i.e. we did not take splice variants into account). From these assemblies, orthologous genes were extracted using HaMStR^68^ and based on a defined ortholog set assembled from six arthropods: *Daphnia pulex*, *Acyrthosiphon pisum, Pediculus humanus*, *Bombyx mori*, *Apis mellifera*, and *Drosophila melanogaster*. For this, and all subsequent analyses, the two sampled populations of *M.* nov. sp. 2 (Mt. Arthur and Sewell Peak, Fig. 1 and Supplementary Tables 1, 2) were analysed separately because they are genetically divergent and possess different mitochondrial DNA backgrounds^23^. After filtering results to exclude genes with more than 2% missing data, the remaining 274 genes (200,379 base pairs (bp)) were aligned using MAFFT v.7.047beta^69^, concatenated, and used in phylogeny construction. Concatenating the sequences made analysis of this large dataset possible, it is also unlikely to affect the species-level relationships we are interested in here^70^,^71^. Phylogenetic trees were constructed from three independent analyses: Bayesian nucleotide (partitioned by codon position) and amino acid analyses conducted using MrBayes v3.2.1^72^,^73^, and likelihood analyses of nucleotide sequences generated with Garli 2.0^74^.

### Differential gene expression

Expression counts were generated by mapping cleaned read to the *de novo* assemblies using Bowtie2^75^ and then extracting reads counts using HTSeq-count^76^. Differentially expressed genes were identified using the combined results from three statistical packages implemented in R^77^: edgeR^40^ (p < 0.05), baySeq (Li < 0.7)^42^, and DESeq^41^ (p < 0.1). Different statistical cut-offs were chosen to encapsulate the different levels of stringency among packages. To compare our results to cold-associated expression in other insect systems^36^,^37^,^38^,^39^, we examined expression levels in a set of *a priori* candidate genes based on their best Blastx hit: genes relating to carbohydrate metabolism^36^(sorbitol dehydrogenase, glucose 6 -phosphate isomerase, glyceraldehyde-3-phosphate dehydrogenase, glucose 6 -phosphate isomerase, glyceraldehyde-3-phosphate dehydrogenase, glucose 6 phosphate dehydrogenase, ribose 5-phosphate isomerase, “carbohydrate”), heat shock proteins^37^, smp-30^38^, a suite of candidate genes based on previous work in Drosophila (UDP-galactose 4’-epimerase, Trehalase, desaturase 1, desaturase 2, Serotonin transporter, Ecdysone-induced protein 28/29 kD, Eip71CD, MLF1-adaptor molecule Pyrroline 5-carboxylate reductase, shibire, DnaJ-like-1, Cytochrome b5-related, Serotonin receptor 7, eyeless and period^39^, and any genes containing “circadian”, “cold” and “freeze” (Full results in Supplementary Data 2). GO ontology assignment^44^ and functional enrichment for these terms using a Fishers exact test were implemented in Blast2GO^45^, conducted separately within each species.

### Tests for selection

To test for evidence of positive selection on structural cuticular genes, we first generated a set of alignments for all orthologs from this GO category. We used reciprocal best-hits blast, followed by manual curation, to identify orthologous structural cuticular genes (or partial genes) that were present in at least five datasets. Not all genes were significantly differentially expressed in our data (Fig. 3); we have labelled them Orth 1–17 (Fig. 3 and Supplementary Table 6). Tests for selection were implemented in the CODEML package of PAML^78^. Two sets of site models (M1a vs. M2a and M7 vs. M8) were compared to identify amino acid sites under selection. To test for positive selection in response to cold adaptation, branch-site models were conducted separately using four different branches as foreground lineages: the branches leading to *N. annulata*, *T. salebrosus*, *Micrarchus* and *S. acornutus* (Fig. 1). The first three branches were chosen to detect lineage- and species-specific adaptation associated with montane cold survival, while tests on the *S. acornutus* branch allowed for a comparison between this divergent lineage with poor cold tolerance and its monophyletic sister group comprising all other NZ genera. To visualize these results, heat maps were generated for all putative orthologs. These depict the log_2_ fold change, as calculated by DESeq, and significant results from selection tests; the full results from these analyses are in Supplemental Table 6.

## Additional Information

**How to cite this article**: Dennis, A. B. *et al.* Parallel molecular routes to cold adaptation in eight genera of New Zealand stick insects. *Sci. Rep.* **5**, 13965; doi: 10.1038/srep13965 (2015).

## Supplementary Material

Supplementary Methods and Tables

Supplementary Dataset 1

Supplementary Dataset 2

Supplementary Dataset 3

Supplementary Dataset 4

## Figures and Tables

**Table 1:** Summary of Illumina sequencing for analysis of differential gene expression.

Species	Collection locale	*n* individuals		Differentially Expressed Genes	
		Cold shock	Control	Upregulated	Downregulated
*Micrarchus hystriculeus*	Paengaroa (c)	3	3	805	646
*Micrarchus* nov. sp 2	Mt. Arthur (a)	6	6	828	781
*Micrarchus* nov. sp 2	Sewell peak (j)	3	3	8143	9348
*Niveaphasma annulata*	Dunedin (g)	3	3	864	1723
*Niveaphasma annulata*	Remarkables (f)	3	3	902	385
*Asteliaphasma jucundum*	Waitakere Ranges (k)	3	3	1028	460
*Tectarchus salebrosus*	Port Hills (m)	3	3	654	532
*Tectarchus ovobessus*	Paengaroa (c)	3	3	629	282
*Argosarchus horridus*	Paengaroa (c)	3	3	445	508
*Clitarchus hookeri*	Auckland (n)	3	3	1176	1617
*Acanthoxyla sp.*	Auckland (n)	3	3	315	269
*Spinotectarchus acornutus*	Waitakere Ranges (k)	3	3	662	519

Each biological replicate came from a single individual. Letters next to collection locales correspond to map locals in Fig. 1(B). Cold shock treatment was for one hour at −5 °C, followed by 1 hour recovery. Full collection details are in Supplemental Table 2, and details of sequencing results are in Supplemental Table 5 and Supplemental Data 1. Differentially expressed genes are the combined results from three statistical packages: EdgeR (p < 0.05), DESeq (P < 0.1), and BaySeq (Li > 0.7).
