# Supplementary material for: Parallel molecular routes to cold adaptation in eight genera of New Zealand stick insects
Source: Sci Rep. 2015 Sep 10;5:13965. doi: 10.1038/srep13965 (PMC4564816; doi:10.1038/srep13965)
Supplement: Supplementary Methods and Tables [file srep13965-s1.pdf]

Title: Parallel molecular routes to cold adaptation in eight genera of New Zealand stick insects

Authors: Alice B. Dennis[a,b,c,d*], Luke T. Dunning[a,b,e,f], Brent J. Sinclair[g], Thomas R. Buckley[a,b,e]

SUPPLEMENTARY MATERIAL

Details of Supplementary Information

Supplementary Information

Supplementary Methods: More detailed descriptions methods in the main manuscript.

Supplementary Table 1: Summary of species ranges, reproductive mode, and collection sites.

Supplementary Table 2: Full collection details for both RNA-seq and Physiology tests.

Supplementary Table 3: Summary of physiological testing.

Supplementary Table 4: Location and dates placed of environmental data loggers.

Supplementary Table 5: Summary of RNA-seq sequence data and de novo assemblies.

Supplementary Data 1: Summary of differential expression results from 3 statistical packages

Supplementary Data 2: Summary of *a priori* genes in all datasets, chosen based on best blast hit. Including, differential expression results and full details of blast results.

Supplementary Data 3: Summary of Fisher's Exact tests, and lists of all significantly differentially expressed GO terms for each species

Supplementary Data 4: Details of cuticular genes depicted in Fig. 3 and their blast results.

Supplemental Methods

These supplementary methods include additional information to the methods detailed in the main article. Citation numbers refer to the main article, and additional citation are named in text and listed alphabetically at the end of this section (Supplementary References).

<u>Collections</u>

Insects were collected at sites throughout New Zealand between January and April of 2011 and 2012. Locales, altitudes and Crosby Codes (Crosby *et al.* 1998) for all collections are in Tables S1 and S2, and are mapped in Figure 1. Collections were made by either finding individual animals by eye or by beating vegetation. In all cases we used only mature, adult females. Animals whose RNA was extracted for Illumina sequencing were transported to Auckland in insulated containers and maintained in the lab at room temperature with an approximately 12:12 h light:dark cycles prior to treatment. During this time, insects were feed *ad libitum* on fresh *Rubus sp.*, *Muehlenbeckia sp.*, *Metrosideros excelsa*, *Lophomyrtus bullata* or *Hoheria sp.*, and were regularly misted with fresh water. For physiological trials, animals were transported live to the University of Otago in insulated containers and maintained for a minimum of seven days prior to treatment. Throughout these trials, they were maintained under controlled conditions at 18˚C under 13:11 h light:dark cycle and fed *Rubus sp. ad libitum*.

<u>Environmental Temperatures</u>

Data loggers (iButton thermochron data loggers; CD1992L, Maxim-Dallas Semiconductor) were housed in rain covers made of c.40 mm deep plastic cups open at one end and placed at each collecting site to measure temperature at 1.5 h intervals during at least one winter season (Fig. 2, Supplementary Table 4 and detailed in Ref 24). Where leaf litter >10 cm deep was

present, loggers were placed in both the litter and in the canopy. Leaf litter has been shown to buffer temperature in these habitats and reduce the number of freezing events[24], but these differences are not as great as those seen between sites. Thus, to broadly compare temperatures experienced at each site, we averaged all data loggers at each site. To depict winter lows in Figure 2, boxplots were constructed from all data recorded in the winter months (June- August). All calculations were performed in R[68].

Cold tolerance measurements: SCP and 6 h frozen

We determined the temperature at which ice formation was spontaneously initiated (the supercooling point SCP) in n= 105 individuals of ten species (n=4 - 40 per species, Supplementary Tables 2, 3). A 30-gauge Type-K thermocouple (Omega, Laval, QC, Canada) was held against the thorax of each individual between the middle and hind legs with a piece of flexible polymer putty (Blue Tack, Bostik, Auckland, New Zealand). The insect was then placed in a glass tube (ID 2cm) in a double-jacketed acrylic chamber cooled by a methanol-water mix circulated from a refrigerated circulator. The insects were held at +5 °C until their body temperature stabilized, and were then cooled at 0.25 °C/min. The thermocouple was connected to an electronic thermometer (HH309A, Omega), which interfaced to a computer which recorded data at 1 s intervals. The SCP was identified as the lowest temperature reached by each individual before the beginning of the exotherm (a measurable temperature rise) indicating the release of the latent heat of crystallization and freezing. Individuals were removed from the glass tube to room temperature (c. 20 °C) within 30 s of the initiation of freezing, marked individually and returned to plastic containers (1 L) for monitoring; the chamber was designed for individual removal upon freeze onset so that the period of freezing was not longer for some individuals, nor were neighbours disturbed by this removal. During this period, insects were maintained under the same controlled conditions and fed *Rubus sp.*

ad libitum. Survival was assessed after seven days and used to indicate whether individuals could survive a small amount of internal ice formation.

Because it can take several hours for ice formation to reach equilibrium in insects[51], we determined whether individuals of each species could survive 6 h of the progression of internal ice formation (Figure 2, Supplemental Table 2, 3). Individuals (n=4 per species per run) were held in contact with a thermocouple and placed in a glass tube in a double-jacketed acrylic chamber cooled with circulating methanol-water solution, as above. Insects were held at +5 °C and then cooled at 0.25 °C/min until the initiation of freezing in the first individual; these batches were grouped by species and all individuals froze within a relatively short period (<5 minutes). The temperature ramp on the refrigerated circulator was then halted (the temperature of the stick insects then continued to decline for a few minutes), and insects were held at that temperature for 6 h before rewarming at 0.25 °C/min. Survival was monitored as above. During these runs, 93 of the 95 individuals we tested froze; the two exceptional individuals were *M*. nov. sp. 2 collected from Mt. Arthur and were excluded from further analyses.

To calculate the SCP for each locale/species, we used both the specific tests to measure supercooling point and the supercooling points observed during the 6 h trials, but only in instances where supercooling was observed during the bath’s temperature decline.

Cold treatments for RNA-seq

Cold-chock treatments of all individuals used in RNA sequencing were conducted at Landcare Research, Auckland, New Zealand. Cold treatments were conducted in a Sanyo MIR-154 (Sanyo Scientific, Bensenville, IL, USA) cooled incubator and have been previously described by Dunning *et. al.*[21]. In all treatments, stick insects were placed in empty, dry plastic containers and left to settle for about one hour. Cold shocked individuals

were placed in a +21 ˚C incubator and held for 10-15 minutes prior to cooling to -5 ˚C at approximately 1 ˚C per minute. The one hour treatment began when the air temperature in the incubator reached -5 ˚C, and was followed by a period of warming to +21 ˚C. Once the incubator had reached +21 ˚C, insects were held for one hour before immediate snap freezing at -80 ˚C freezer. Control individuals were treated on the same day by placing containers in the incubator at 21 ˚C for two hours before immediate freezing at -80 ˚C.

RNA extraction and library preparation

All samples were stored at -80 ˚C until extraction. For all species, cDNA libraries were constructed from between 6 and 12 individual stick insects (Supplement Table 5). RNA was extracted from the head, prothorax and antennae only. Tissue was ground in liquid nitrogen and extracted using Trizol reagent (Invitrogen, Carlsbad, CA, cat #15596) according to the manufacturer's instructions, with the following modifications: ground samples were incubated overnight at room temperature in Trizol, and ethanol precipitations were incubated overnight at +4 ˚C. Products of Trizol extraction were subsequently spin column purified using the RNeasy mini kit (Qiagen, Venlo, Netherlands, cat # 74104). Purified total RNA was verified on an Agilent 2100 Bioanalyzer (Agilent Technologies, Santa Clara, CA, USA) and quantified with a Nanodrop 2000 spectrophotometer (Thermo Scientific, Wilmington, DE, USA).

All cDNA libraries were prepared using the Illumina TruSeq RNA kit (v1/ Cat# FC-121-1001 for *Micrarchus*[21], v2/ Cat # RS-122-2001 for all other species) with an input of approximately 2.5 µg of total RNA. In all cases, the PCR amplification step of the protocol was performed for 12 cycles. Library quality was verified using a Bioanalyzer prior to sample quantification and sequencing at the High Throughput Genomics Core Facility at the Huntsman Cancer Institute, University of Utah, with 8 – 18 individuals per flow cell lane.

Sequence clean-up and assembly

Prior to assembly, raw Illumina reads were filtered to retain only high quality sequences in several steps. To remove sequences more likely to contain errors, all reads containing an 'N' were discarded using ShortRead v1.16.3[55] implemented in R. Low quality (phred score < 30) and Illumina primer sequences were then trimmed using Cutadapt v1.1[53]. Lastly, polyT stretches on sequence ends were trimmed using PRINSEQ lite v 0.6[54] and remaining reads shorter than 25bp were discarded. The quality of each individual library before and after clean-up was verified using FastQC v 0.10.1[56].

For each species, *de novo* assembly was conducted separately using Trinity v10.05.2012[57] with the default settings and the -REDUCE flag. The two populations of *M.* nov sp. 2 (Mt. Arthur and Sewell Peak) were aligned separately because of their higher levels of divergence and different mitochondrial DNA background[21]. The alignment for *N. annulata* was jointly constructed using 6 individuals from the Remarkables, 6 individuals from Dunedin, and 9 individuals from Nevis Rd. (manuscript containing these data in prep). In all, this resulted in 11 *de novo* transcriptome assemblies, each constructed from between 6 and 21 individuals (Supplemental Tables 5).

To reduce an apparent overabundance of called splice variants, the resulting transcripts were clustered using CD-HIT-EST[58], which combined all contigs with > 95% similarity. During library preparation, removal of ribosomal RNA could be both incomplete and unequal among samples. To remove this bias among libraries, ribosomal sequences were removed by Ribopicker online[59]. However, not all sequences were removed and in some cases 12S, 16S and 28S were manually identified by blast and removed from the assembly. The resulting number of contigs, as well as their N50 and N90, are listed in Supplemental Table 5.

Assemblies were annotated by a BlastX[60] sequence search (e-value threshold $1e^{-10}$) against the National Centre for Biotechnology Information (NCBI) non-redundant (*nr*) database. Approximately 10% of all contigs were annotated this way, but that number was higher (~25%) when only contigs > 200 bp were retained. However, to maximize comparisons among datasets, 100-200bp long contigs were retained in all analyses. Gene ontology (GO) terms were assigned using Blast2GO[38] based on the BlastX results.

To determine the overall similarity among the transcripts sequenced across species, pairwise blasts with $1e^{-10}$ e-value threshold were conducted among each of the Trinity alignments. Based on this, all alignments shared more than 99% of their transcripts with one another (results not shown). To search for *a priori* genes (Supplementary Dataset 3), we assembled a list of candidate genes from several sources[35-38] and retrieved all matches to these in the top blast hit from the Blastx results (above).

<u>Phylogenetic relationships among NZ stick insects</u>

Orthologs used in phylogenetic analyses were extracted from each species' Trinity assembly using HaMStr[61], which was kindly run by I. Ebersberger using a core ortholog set containing 2,423 orthologs built based on reference genomes in six arthropod taxa: *Daphnia pulex*, *Acyrthosiphon pisum*, *Pediculus humanus*, *Bombyx mori*, *Apis mellifera*, and *Drosophila melanogaster*. Recovery of orthologs was high across taxa; 2,336 of core orthologs were detected in at least one dataset. Of these, 1,485 were found in all 11 datasets. However, many of these genes were not full length across all taxa, so we retained only genes which were missing less than 2% of their nucleotides (due to both gaps and missing data). The remaining genes were aligned using MAFFT v.7.047beta[62] with a maximum of 1000 iterations and the --localpair setting. This resulted in 274 genes (200,379 base pairs, bp) that were concatenated used for phylogenetic analysis.

These 274 genes were concatenated, providing a total 200,279 bp for analysis. Concatenating the sequences made analysis of this large dataset possible, it is also unlikely to affect the species-level relationships we are interested in here[65,66]. Bayesian phylogenies were constructed separately for both nucleotide sequences, partitioned by codon, and amino acid sequences using Markov-chain Monte Carlo (MCMC) sampling in MrBayes v. 3.2.1[63,64] for 3 million generations, sampled every thousand. A relative burn-in of 25% was used with the following priors: exponentially distributed substitution rates, unconstrained branch lengths, empirically estimated state frequencies, exponential gamma shape parameter set to five for among-site rate variation and proportion of invariable sites uniformly distributed between zero and one. Each data set was analysed in three independent runs for both the nucleotide and amino acid dataset. Likelihood bootstrap values were generated in Garli[65] using a partitioned model with separate GTR+I+G models for each codon position and 100 bootstrap replicates with one search replicate per bootstrap.

<u>Differential expression analysis of Illumina data</u>

As in all analyses, these calculations were performed separately for each Trinity alignment. To generate expression counts for each Trinity contig, raw reads for each individual were mapped back onto the assemblies using Bowtie2[66] with the '-sensitive' configuration. Raw counts were generated for each individual using HTSeq-count[67], run against .gff files generated by Cufflinks (Trapnell *et al.* 2012). Calculations of differential expression were similar to previously published methods[21]. Differentially expression genes were identified using three packages implemented in R. First, edgeR[30] was run using tagwise dispersal estimates and a cut-off of $p < 0.05$. Second, baySeq[32] was run using the corrected counts generated by edge R and a cut-off of likelihood < 0.7. Lastly, DESeq[31] was run using recommended settings and cut-off of $p < 0.1$. In all cases, contigs were only considered for

differential expression analysis if they had at least one count per million mapped reads and were present in at least half of the samples (three individuals in all cases except *M.* nov. sp. 2 Mt Arthur, where was six control and 6 treatment insects were measured). The resulting lists of significantly differentially expression genes for each species (Supplement Data 2) was compiled by combining the results from each package, and removing redundant contigs (produced by more than one package). Contigs with infinite fold change were retained (i.e. those with zero counts in all members of one treatment). There was generally more overlap among the outputs of baySeq and edgeR than either package with DESeq. Enrichment for GO terms was tested using a Fisher's exact test, implemented in Blast2GO. In this, we tested for GO terms associated with the genes significantly up-regulated in the cold against all other genes identified in the transcriptomes (Supplementary Data 3).

<u>Orthologous cuticle genes and tests of selection</u>

Based on the results of the Fisher's exact test, genes in the GO category "structural constituent of cuticle" were the only annotated genes to be significantly (FDR < 0.05) differentially expressed in more than one species. To further investigate this, we extracted all genes annotated to this GO category from the individual *de novo* transcriptome assemblies (based on their GO assignment in Blast2GO). To identify orthologs, pairwise comparisons were made between these "structural constituent of cuticle" genes from each dataset using reciprocal blast and manual curation. Particular attention was paid to sorting orthologs with the same annotation. Each ortholog was aligned in Geneious (Geneious version 6.1.7) using ClustalW (Larkin *et al.* 2007), and the alignments were then manually inspected, phased, and trimmed of untranslated regions (UTR). Despite their groupings using reciprocal blast, several sequences were obviously not orthologous and either some individuals or the entire sequence was removed from the alignment. Genes were also not retained if they were present

in less than 5 species, if they were < 200bp in length, or if they could not be phased. For the remaining 17 genes, now arbitrarily named Orth 1 -17, we generated a heatmap (Figure 3) depicting the log_2fold change of that gene each species, as calculated by DESeq.

To detect patterns of selection on structural cuticular genes, alignments of orthologous genes were analysed in the CODEML package of PAML[42]. For all comparisons, we used the phylogenetic tree produced by the 274 genes described above (Figure 1); in cases with fewer than the full 11 taxa, missing branches were removed and an unrooted tree was exported in Mesquite (Maddison& Maddison 2011). We implemented two separate sets of analyses to look for patterns of selection in these genes across the phylogeny of NZ stick insects. To detect amino acid sites under selection, we compared two site models: M1a vs. M2a and M7 vs M8. To identify positive selection on amino acids within a particular lineage, we implemented the branch-site model test in four different independent runs. Each run was starred one of the following different foreground branches: to test for selection associated with occurrence in montane and colder high latitudes, we tested (1) the *Micrarchus* clade, (2) the *N. annulata* branch, and (3) the *T. salebrosus* branch. In association with colonization of cooler habitats we tested (4) the *S. annulata* branch (separating *S. annulata* from all other species). In all cases, the Bayes Empirical Bayes (BEB) method was used to identify sites under selection.

Supplementary References

Crosby TK, Dugdale JS, Watt JC (1998) Area codes for recording specimen localities in the New Zealand subregion. *N.Z. J. Zoo.* **25**, 175-183.

Geneious (version 6.1.7) Geneious v6.1.7 created by Biomatters. Available from http://www.geneious.com/.

Larkin MA, Blackshields G, Brown NP, *et al.* (2007) Clustal W and Clustal X version 2.0. *Bioinformatics* **23**, 2947-2948.

Maddison WP, Maddison DR (2011) Mesquite: a modular system for evolutionary analysis. Version 2.75 http://mesquiteproject.org.

Trapnell C, Roberts A, Goff L, *et al.* (2012) Differential gene and transcript expression analysis of RNA-seq experiments with TopHat and Cufflinks. *Nat. Protocols* **7**, 562-578.

Species		Collection locale					
Species	Geographic range summary	Locale	Crosby code	Reproductive mode	Latitude	Longitude	Altitude
North							
Spinotectarchus acornutus	Northern NI, lowland habitats [a]	**Waitakere** Ranges, Auckland	AK	Sex	36° 52.1	174° 28.0	0-474
Asteliaphasma jucundum	Northern NI, lowland habitats [a]	**Waitakere** Ranges, Auckland	AK	Sex	36° 52.1	174° 28.0	0-474
Clitarchus hookeri	Lowland throughout NI & eastern SI. Obligate parthenogenic populations more common in the south [16]	**Auckland** Botanical Gardens	AK	Sex	37° 0.0	174° 54.8	75-100
Acanthoxyla sp.	Lowland NI & SI. Obligate parthenogen [76]	**Auckland** Botanical Gardens	AK	Asex	37° 00.4	174° 54.2	75
Tectarchus ovobessus	Mid to southern NI and northern SI. Lowland to 1000 m a.s.l. [a]	**Paengaroa** Scenic Reserve, Taihape	RI	Sex	39° 38.8	175° 42.6	517-540
Argosarchus horridus	Lowland NI & SI. Obligate parthenogenic populations more common in the south [17]	**Paengaroa** Scenic Reserve, Taihape	RI	Sex	39° 38.8	175° 42.6	517-540
Micrarchus hystriculeus	Southern NI & northern SI. Lowland to 970 m a.s.l. [20]	**Paengaroa** Scenic Reserve, Taihape	RI	Sex	39° 38.8	175° 42.6	517-540
Micrarchus nov sp 1	Northeastern SI. Lowland to 1100 m a.s.l. [20]	**Puhipuhi** Scenic Reserve, Kaikoura	KA	Sex	42° 14.2	173° 45.1	241
Micrarchus nov sp 2	Obligate to altitudes at or above tree line, found on northwest SI between 640 and 1400 m a.s.l. only [20]	**Mt. Arthur**, Kahurangi National Park	NN	Sex	41° 11.9	172° 42.7	1336-1347
Micrarchus nov sp 2		**Sewell Peak**, Paparoa National Park	BR	Sex	42°24.3'	171°20.6	749-822
Tectarchus salebrosus	Sea level to high altitude 1100 m a.s.l. [a]	Kennedy’s Bush, **Port Hills**, Christchurch	MC	Sex	43° 37.9	172° 37.5	420

Niveaphasma annulata	Mid to southern SI only. Broadly occurs from sea level to above tree line (1,000 m a.s.l.). Some high altitude populations are parthenogenetic [19]	**Ohau**, Mackenzie Basin	MK	Sex	44° 14.5	169° 48.2	780-980
Niveaphasma annulata		**Remarkables**, Otago*	CO	Asex	45° 01.7	168° 47.0	930-1031
Niveaphasma annulata		**Nevis**, Bannockburn, Otago*	CO	Asex	45° 09.3	169° 07.6	904
Niveaphasma annulata		**Dunedin**, Leith Valley	DN	Sex	45° 51.0	170° 30.3	49
Niveaphasma annulata		**Seward Moss**, Southland	SL	Sex	46° 32.5	168° 26.0	9
Niveaphasma annulata		**Papatowai**, Old Coach Rd. Catlins	SL	Sex	46° 33.2	169° 28.5	0
South							

Supplementary Table 1. Summaries of each species' entire ranges and the specific collection locales used in this study. Latitude, longitude and altitude taken from handheld GPS devices, Crosby Codes from Crosby (Supplementary Ref 1). Range summary indicates general features of entire species range. NI = North Island, SI = South Island. Reproductive mode denotes either entirely parthenogenetic populations (asex), or those in which males have been observed (sex). Bracketed referenced refer to those in main text. [a] Records from New Zealand Arthropod Collection, Landcare Research, Auckland, New Zealand

Additional Reference:

[76] Buckley, Thomas R., Dilini Attanayake, Duckchul Park, Shanthinie Ravindran, Tony R. Jewell, Benjamin B. Normark. 2008. Investigating hybridization in the parthenogenetic New Zealand stick insect *Acanthoxyla* (Phasmatodea) using single-copy nuclear loci. Mol Phylogenet Evol. 48:335–349

		Collections for RNA-seq			Collections for Physiology		
Species	Locale	Date collected	Date treated	n, Ilumina	Date collected	n, SCP	n, 6h
**North**							
Spinotectarchus acornutus	**Waitakere** Ranges, Auckland	Jan-Feb 2012	5 Apr 2012	6	Jan- Feb 2012	8	4
Asteliaphasma jucundum	**Waitakere** Ranges, Auckland	Jan- Feb 2012	3 Apr 2012	6	Jan- Feb 2012	8	4
Clitarchus hookeri	**Auckland** Botanical Gardens	9- 11 Feb 2011	15 Feb 2011	6	17 Feb 2012	7	4
Acanthoxyla sp.	**Auckland** Botanical Gardens	10 Apr 2012	12 Apr 2012	6	3-10 April 2012	8	4
Tectarchus ovobessus	**Paengaroa** Scenic Reserve, Taihape	13 Apr 2012	24 Apr 2012	6	23-26 Jan 2012	5	4
Argosarchus horridus	**Paengaroa** Scenic Reserve, Taihape	25 Mar 2011	1 Aug 2011	6	13-15 April 2012	5	4
Micrarchus hystriculeus	**Paengaroa** Scenic Reserve, Taihape	25 Mar 2011	11 May 2011	6	23-26 Jan 2012	4	4
Micrarchus nov sp 1	**Puhipuhi** Scenic Reserve, Kaikoura				15 Jan 2012	4	4
Micrarchus nov sp 2	**Mt. Arthur**, Kahurangi National Park	13 Feb 2011	17 Mar 2011	12	23 Jan 2012	4	6
Micrarchus nov sp 2	**Sewell Peak**, Paparoa National Park	11 Feb 2011	17 Mar 2011	6			
Tectarchus salebrosus	Kennedy's Bush, **Port Hills**, Christchurch	23 Feb 2012	3 May 2012	6	23 Feb 2012	8	4
Niveaphasma annulata	**Ohau**, Mackenzie Basin				25-26 Feb 2012		4
Niveaphasma annulata	**Remarkables**, Otago*	27 Feb 2011	25 Mar 2011	6	28 Feb 2012		7
Niveaphasma annulata	**Nevis**, Bannockburn, Otago*				27 Feb 2012	8	4
Niveaphasma annulata	**Dunedin**, Leith Valley	4 Mar 2011	25 Mar 2011	6	3 Mar 2012	8	6
Niveaphasma annulata	**Seward Moss**, Southland				29 Feb 2012	8	4
Niveaphasma annulata	**Papatowai**, Old Coach Rd. Catlins				1 Mar 2012	8	4
South							

Supplementary Table 2. Full collection details of all samples used in both RNAseq following cold shock and physiological tests.

Species	Locale	Supercooling point (SCP)	SCP survival (%)	Number SCP	6 h freeze survival (%)	Number 6 h freeze
Spinotectarchus acornutus	Waitakere Ranges	-4.4	38	8	0	4
Asteliaphasma jucundum	Waitakere Ranges	-3.7	88	8	0	4
Clitarchus hookeri	Auckland	-2.9	71	7	0	4
Acanthoxyla sp.	Auckland	-4.4	75	8	0	4
Tectarchus ovobessus	Paengaroa	-3.8	80	5	0	4
Argosarchus horridus	Paengaroa	-4.8	80	5	0	4
Micrarchus hystriculeus	Paengaroa	-5.6	50	4	25	4
Micrarchus nov sp 1	Puhipuhi	-5.8	0	4	0	4
Micrarchus nov sp 2	Mt. Arthur	-4.4	100	4	33	6
Tectarchus salebrosus	Port Hills	-4.5	100	8	75	4
Niveaphasma annulata	Ohau	-2.8	n.d.	n.d.	75	4
Niveaphasma annulata	Nevis	-2.6	88	8	75	4
Niveaphasma annulata	Dunedin	-4.8	100	8	0	6
Niveaphasma annulata	Remarkables	-2.4	n.d.	n.d.	43	7
Niveaphasma annulata	Seward Moss	-2.7	75	8	100	4
Niveaphasma annulata	Papatowai	-3.8	100	8	100	4

Supplementary Table 2: Summary of physiological measures of supercooling poing (SCP) and survival of 6 hours frozen (6hr). Survival was recorded for up to 7 days (7d); 1 indicates survival, 0 indicates death. Supercooling points reported in Figure 2 include both SCP and 6 h tests where freezing occurred during the temperature decline.

**Location**	**Placement**	**Number ibuttons**	**Species present**	**Elevation (m)**	**Date placed**	**Date removed**	**Latitude**	**Longitude**	**Mean Temp. (ºC)**	**Min. Temp. (ºC)**
North										
Matuku Reserve, **Waitakere** Ranges	Canopy and Litter	5	*Asteliaphasma, Spinotectarchus, Clitarchus*	0-474	2/04/2012	4/09/2013	36° 52.1 S	174° 28.0 E	13.1	-0.4
Huia, **Waitakere** Ranges	Canopy (3) and Litter (3)	6	*Asteliaphasma, Spinotectarchus, Clitarchus*	0-474	9/5/2011 & 2/4/2012	4/09/2013	36° 52.1 S	174° 28.0 E	14.5	0.6
**Auckland** Botanical Gardens	Canopy (4) and Litter (1)	5	*Acanthoxyla*	75-100	27/04/2012	4/11/2013	37° 0.0 S	174° 54.8 E	14.1	-2.4
**Paengaroa** Scenic Reserve, Taihape	Canopy (3) and Litter (3)	6	*Acanthoxyla, Argosarchus, Clitarchus, Micrarchus hystriculeus, Tectarchus huttoni*	517-540	27/3/2011	3/06/2013	39° 38.8 S	175° 42.6 E	8.3	-5.5
**Puhipuhi** Scenic Reserve, Kaikoura	Canopy (3) and Litter (3)	6	*Micrarchus* nov. sp. 1	241	19/7/11	14/01/2012	42° 14.2 S	173° 45.1 E	9.6	-2.5
**Mt. Arthur**, Kahurangi National Park	Canopy (3) and Litter (3)	6	*Micrarchus* nov. sp. 2	1336-1347	15/2/11	22/1/12	41° 11.9 S	172° 42.7 E	5.2	-5.5
**Sewell Peak**, Paparoa National Park	Canopy (3) and Litter (3)	6	*Micrarchus* nov. sp. 2	749-822	13/2/11	26/1/12	42°24.3 S	171°20.6 E	7.5	-6.0
Kennedy's Bush, **Port Hills**, Christchurch	Canopy and Litter	4	*Tectarchus salebrosus, Argosarchus*	893	24/02/2012	2/02/2013	43° 37.9 S	172° 37.5 E	9.8	-2.5

**Ohau**, Mackenzie Basin	In plant near ground	3	*Niveaphasma annulata*	780- 980	28/02/2011	25/02/2012	44° 14.5 S	169° 48.2 E	9.1	-6.0
**Remarkables**, Otago	In plant near ground	3	*Niveaphasma annulata*	930-1031	28/02/2011	3/03/2013	45° 01.7 S	168° 47.0 E	8.7	-5.0
**Nevis**, Bannockburn, Otago	Canopy (3) and Litter (3)	6	*Niveaphasma annulata*	904	28/02/2011	3/03/2013	45° 09.3 S	169° 07.6 E	7.5	-8.0
**Dunedin**, Leith Valley	In plant near ground	2	*Acanthoxyla, Argosarchus, Clitarchus, Niveaphasma*	49	5/03/2011	14/02/2013	45° 51.0 S	170° 30.3 E	9	-0.9
**Seward Moss**, Southland	Canopy (3) and Litter (3)	6	*Niveaphasma annulata*	9	1/03/2011	3/03/2013	46° 32.5 S	168° 26.0 E	9.5	-11.6
**Papatowai**, Old Coach Rd., Catlins	Canopy (3) and Litter (3)	6	*Niveaphasma annulata*	0	4/03/2011	3/03/2013	46° 33.2 S	169° 28.5 E	9.3	-2.9
South										

Supplemental Table 4: Location and placement dates of ibutton data loggers, and species that occur at that locale. Temperature was measured every 1.5 hours.

**Species**	**Locale**	**Number of Individuals**	**Number of Clean Reads**	**Clean *de novo* contigs**	**Longest Contig**	**N50 (bp)**	**N90 (bp)**
Spinotectarchus acornutus	Waitakere Ranges	6	57,145,451	100,486	25,298	389	120
Asteliaphasma jucundum	Waitakere Ranges	6	56,981,479	88,628	17,151	467	122
Clitarchus hookeri	Auckland	6	58,148,764	84,307	23,840	519	123
Acanthoxyla sp.	Auckland	6	67,462,015	122,965	26,075	472	121
Tectarchus ovobessus	Paengaroa	6	64,093,476	105,278	26,532	618	125
Tectarchus salebrosus	Port Hills	6	55,285,976	70,268	10,789	519	123
Argosarchus horridus	Paengaroa	6	62,510,873	86,970	25,983	588	125
Micrarchus hystriculeus	Paengaroa	6	56,471,214	116,516	16,697	370	118
Micrarchus nov. sp. 2	Mt. Arthur	12	118,263,415	177,668	14,290	439	120
Micrarchus nov. sp. 2	Sewell Peak	6	59,833,254	124,050	14,995	414	120
Niveaphasma annulata	Remarkables	6	71,340,806	193,845*	37,991	581	122
Niveaphasma annulata	Dunedin	6	60,767,621				

Supplemental Table 5: Summary of Illumina sequencing of cDNA libraries and separate *de novo* assemblies of these for each species. "Clean *de novo* Contigs" are the results of individual alignments in Trinity and subsequent processed (see Methods). *Joint *Niveaphasma* alignment includes both locales reported here (Dunedin and Remarkables), plus 94,655,424 reads from 9 individuals collected at Nevis Rd. (manuscript in prep.).

SITE MODELS												BRANCH SITE MODELS							
						2Δl				Sig aa sites (BEB)		*Spinotectarchus*		*Micrarchus*		*Niveaphasma*		*T. salebrosus*	
**Gene**	**bp**	**n Taxa**	**ө**	**k (M0)**	**ω (M0)**	**M0:M3**	**M1a: M2a**	**M7:M8**	**M8:M8a**	**M8**	**M2 a**	**2Δl**	**Sig aa sites (BEB)**	**2Δl**	**Sig aa sites (BEB)**	**2Δl**	**Sig aa sites (BEB)**	**2Δl**	**Sig aa sites (BEB)**
Orth15	1083	11	0.03	2.24	0.27	48.32**	3.00	3.92	2.94	3*		0	0	14.87**	1*	0	0	0	0
Orth1	162	7	0^2	1E-04	1.0E-04	0	0	0	5.7E-04	0				2.40E-04	0	0	0	0	0
Orth2	126	7	0.03	13.45	0.09	4.17	0.00	3.6E-05	0	0				0	0	4.203*	2*	0	0
Orth16	119	5	0.02	7.51	0.55	0	0	3.4E-05	0	0		0.000	0	3.00E-05	0				
Orth3	104	9	0.05	2.55	0.04	0	1.5E-04	1.5E-04	2.0E-04	0		0	0	0	0	0	0		
Orth17	444	7	0.04	2.20	0.34	39.53**	8.76**	8.90*	8.70*	2**/*	2*			0	0	0	0	0	0
Orth4	282	11	0.03	2.62	0.06	14.34*	0	1.62	0	1		3.179^x	1*	0	0	0.339	1*	1.61	0
Orth5	441	7	0.02	9.07	0.06	26.71**	6.38*	8.69*	6.81**	1**	1**			0	0	4.029*	1*		
Orth6	414	9	0.04	3.11	0.35	32.60**	11.16**	11.22**	11.22**	1**	1**			2.590	0	0	0	0	0
Orth7	267	6	0.02	2.92	0.04	3.00	2.8E-03	5.6E-04	5.0E-04	0				0	0	0	0		
Orth8	412	8	0.05	3.05	0.07	50.80**	6.33*	2.23	1.35	1**	1**			0.883	0	0	0	0	0
Orth9	312	8	0.03	5.76	1.90	0.59	3.45	3.45	3.45	0				0	0	4.091*	0	0.399	0
Orth10	372	9	0.03	5.27	0.08	5.04	0	1.2E-04	0	0				0	0	0.080	0	0	0
Orth11	258	11	0.02	1.60	0.04	10.69*	8.0E-06	0.81	1.6E-05	0		0	0	0	0	0	0	0	0
Orth12	120	6	0.07	1.62	0.18	2.36	0.00	0.05	0.05	0		3.715^x	1**	0	0	0	0		
Orth13	63	5	0.03	2.06	0.09	0	2E-05	2E-05	2E-05	0		0.000	0	0	0			0	0
Orth14	124	10	0.77	0.63	0.17	111.77**	2.04	1.02	1.01	1**		0	0	0	0	0	0	0	0

Supplementary Table 6: Summary of Paml (codeml) tests for amino acid sites under selection. Significance: ^{x} P> 90%; * P>95%; ** P>99%. ^{2}: no variable sites in Orth1. **n** = number of species with sequences; **bp** =sequence length; **ө** = nucleotide diversity; **k** = ratio of transitions to transversions; **ω (M0)** = ratio of

synonymous to nonsynonymous substitutions under the M0 model; **2Δl** is given for each model comparison (Site models; MO:M3; M7:M8 and M8:M8a. Branch models; between a tree with a fixed ω across all branches, and one where ω is allowed to change on the focal branch), which is twice the difference between the log likelihood of the two nested site-specific models implemented in PAML. **Sig aa sites (BEB)** = number of amino acids detected under positive selection under a Bayes Empirical Bayes model.
